# Carotid and femoral bruits as cardiovascular risk indicators in a middle-aged Finnish population: A 20-year prospective study

**DOI:** 10.1371/journal.pone.0278901

**Published:** 2022-12-09

**Authors:** Karri Parkkila, Antti Kiviniemi, Mikko Tulppo, Juha Perkiömäki, Y. Antero Kesäniemi, Olavi Ukkola

**Affiliations:** 1 Medical Research Center Oulu, Research Unit of Internal Medicine, Oulu University Hospital, University of Oulu, Oulu, Finland; 2 Department of Physiology, Research Unit of Biomedicine, Faculty of Medicine, University of Oulu, Oulu, Finland; University of Otago, NEW ZEALAND

## Abstract

**Background:**

Effective treatment and prevention of cardiovascular (CV) diseases requires reliable methods of assessing individual CV event risk. Although standardized risk calculators like Systematic Coronary Risk Evaluation (SCORE) are sufficient in most instances, sometimes more specific clinical examination is needed to determine the most optimal intervention and its intensity.

**Aim:**

To study whether carotid and femoral bruits provide prognostic information on CV events, CV mortality and all-cause mortality beyond traditional CV risk factors.

**Methods:**

1045 subjects (49.8% men), aged 51.3 ± 5.97 years were clinically examined in the beginning of 1990’s. The subjects were followed for over 20 years and data on CV events and causes of deaths was collected.

**Results:**

During the follow-up period, 241 (23.1%) of the subjects died and 82 (34.6%) of the deaths were of CV origin. Carotid bruits were a significant risk factor for CV deaths only if subjects with previous CV events were included. After adjusting for age, sex, systolic blood pressure, smoking, diabetes, LDL cholesterol, coronary artery disease and stroke, carotid bruits posed a hazard ratio (HR) (95% confidence interval) of 4.15 (2.39–8.52) p<0.001 for CV deaths. After excluding subjects with previous CV events (after which n = 941) neither carotid nor femoral bruits were statistically associated with CV events or all-cause mortality. Adding carotid or femoral bruits in the baseline risk model with traditional CV risk factors did not improve C-statistic, reclassification, or discrimination of the subjects.

**Conclusions:**

Carotid and femoral bruits do not provide clinically useful information in a pure primary prevention setting. Carotid bruits might be useful in evaluating the overall CV risk in a population where recurrent CV events may occur.

## Introduction

Cardiovascular (CV) diseases are known for their infamous position as the number one cause of mortality globally, and over 18 million people died due to CV diseases in 2019 [1]. Fortunately, age-standardized death rates due to ischemic heart diseases have declined since 1980 [2], which is largely the result of both the reductions in major risk factors as well as the improved use of evidence-based medical therapies [3]. Obviously, the work is not done yet. The evidence that both primary and secondary prevention account for a large part of the decline in coronary artery deaths [3] as well as the evidence that the absolute risk reduction is greater in high-risk individuals [4], is encouraging to continue the research and clinical efforts to recognize individuals that would benefit from preventative actions the most.

Estimating an individual’s overall CV event risk is challenging, not least because of the multiple factors contributing to CV diseases [1]. Therefore, risk calculators such as Framingham Risk Score (FRS) and Systematic COronary Risk Evaluation (SCORE) have been developed to help the clinician in evaluating each patient’s CV risk more accurately. Owing to the strong evidence of these risk calculators in quite accurate CV risk prediction, both American and European guidelines are recommending them in assessing the 10-year CV risk in certain populations [5, 6]. In addition to the established risk calculators, more advanced clinical tools for risk estimation, such as coronary artery calcium score [7, 8] and carotid intima-media thickness [9, 10], have been extensively researched with promising results. In fact, also the use of coronary artery calcium score is considered useful by the American [5] and the European [6] guidelines in patients with borderline risk to refine the risk assessment for the possible preventative interventions.

However, much less is known about simpler clinical tests, such as carotid or femoral bruits detected via auscultation, in CV risk assessment. A meta-analysis published in 2008 concluded that having a carotid bruit poses a two-fold risk for CV death or myocardial infarction compared to those without audible bruits [11]. Of note, the majority of the included studies were from 1980s, and the lack of information and adjustments for other traditional CV risk factors needs to be taken in consideration. Similarly, only few publications exist that have explored to role of femoral bruits in CV risk assessment. So far, femoral bruits have been documented to be significant risk factors for the first coronary event [12], and femoral plaques may be, in fact, more accurate predictors of coronary artery calcification as compared to the more studied carotid plaques [13].

The currently used accessory clinical tool (i.e., coronary artery calcium score) for CV risk assessment exploits ionizing radiation, requires specific technology as well as clinical experience for the interpretation of the images. Therefore, exploring the true potential of the quick, easy and safe auscultation of the vasculature in recognizing patients in higher CV risk is of great clinical significance. The objective of the current study is to elucidate the role of carotid and femoral bruits in predicting CV and all-cause mortality in a prospective study setting with a follow-up period of more than 20 years.

## Materials and methods

### Study population

**Fig 1:**
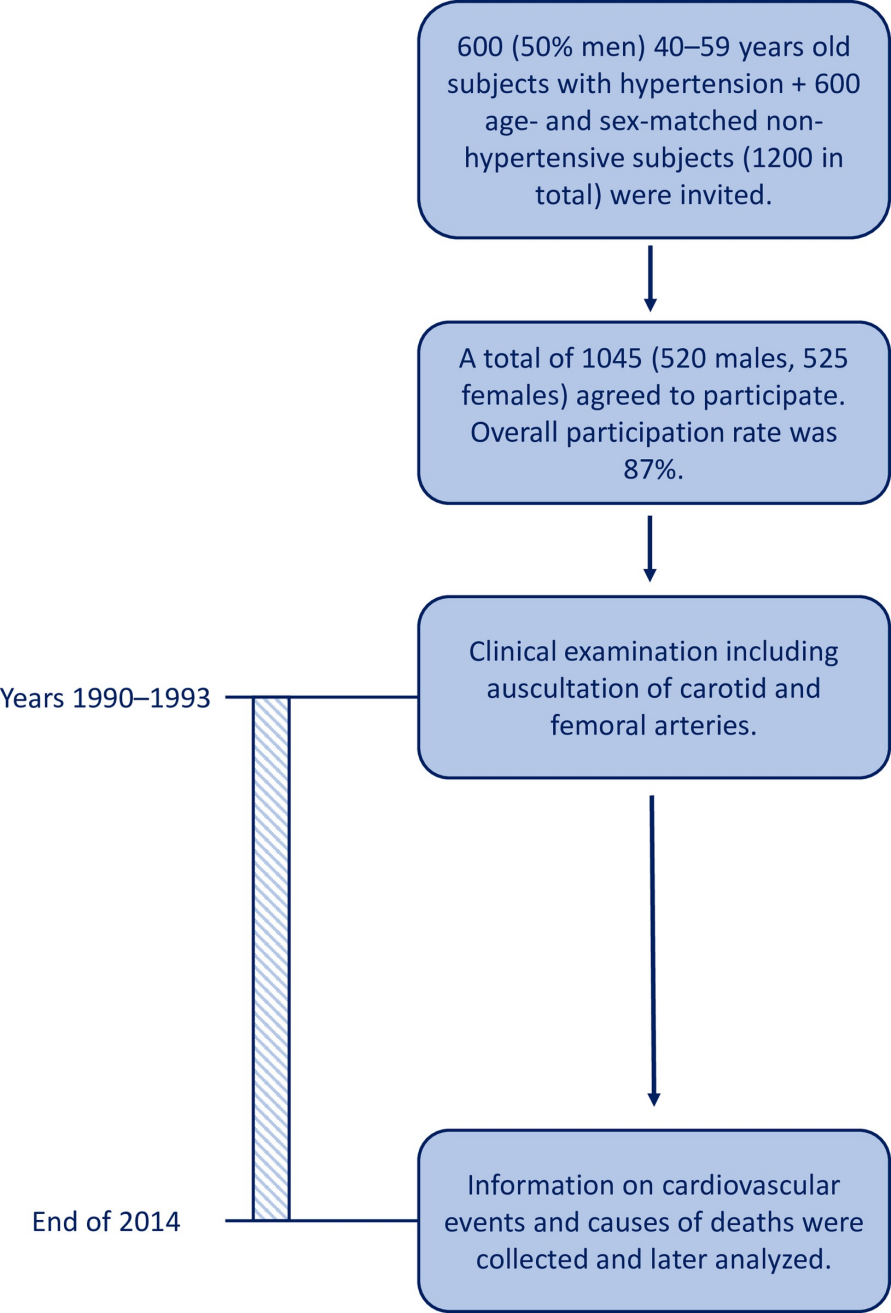
Flow chart of participant recruitment and follow-up.

This study is part of the Oulu Project Elucidating Risk of Atherosclerosis (OPERA) project. The methodologies and recruitment of the study population has been described in previous publications [14–16]. In brief, 600 hypertensive subjects (50.0% men) and 600 age- and sex-matched control subjects were randomly selected from the Social Insurance Institute register to participate in the OPERA study. A total of 1045 subjects (overall participation rate 87.1%), of which 520 were men and 525 were women, participated in the current study. A simple flow chart of participant recruitment and follow-up is presented in Fig 1. The OPERA study was approved by the Ethical Committee of the Faculty of Medicine, University of Oulu and was compatible with the Declaration of Helsinki. Informed consent was obtained from each participant.

### Clinical examination

The clinical examinations took place in the early 1990s. All subjects were examined after 12 h of fasting and each participant was examined during the same time of the day (08.00am-1.00pm). The clinical examination included standard measurements of height, weight, as well as waist and hip circumference. Patients were considered to have a metabolic syndrome based on the International Diabetes Federation criteria (i.e. central obesity plus any two of the following: raised triglycerides, reduced high-density lipoprotein, raised blood pressure, raised fasting plasma glucose) [17]. Blood pressure was measured according to the recommendations of the American Society of Hypertension [18] in a sitting position from the right arm with an oscillometric device (Dinamap® model 18465X, Criticon Ltd., Ascot, UK) after a 10-15-minute rest. Three measurements were made at 1-minute intervals and the means of the last two were used in the analyses. Subjects were considered to be hypertensive if they had systolic blood pressure of ≥ 140 mmHg and/or diastolic blood pressure of ≥ 90 mmHg.

After auscultation of the heart in a sitting position, the physician then bilaterally auscultated the carotid arteries. The auscultation was performed over the course of the carotid artery up to the angle of the jaw with the participant’s head in neutral position. If the physician was uncertain of the findings, the auscultation was repeated. Likewise, the auscultation of the femoral arteries was performed bilaterally from the inguinal ligament downwards about 10 cm distally alongside the natural course of the artery, while the participant was in supine position. Auscultation was repeated if necessary or if the physician was uncertain of the findings. If detected, carotid or femoral bruit was characterized as right, left, or bilateral. Males and females were auscultated by two separate physicians.

In addition to the clinical examinations, a questionnaire covering detailed information about each participant’s smoking habits, alcohol consumption, physical activity, medication, and previous medical history was completed by two specially trained nurses. The presence of coronary artery disease (CAD) and previous stroke or transient ischemic attack (TIA) was determined by interviewing the patient and confirming the diagnosis from the medical records. Smoking habits was classified as: never smoker, current smoker, not anymore (had smoked regularly before but had quit before enrolling in the study), or occasional smoker (under one cigarette per day). Alcohol consumption was calculated as absolute grams of pure alcohol. Leisure time physical activity was classified as: no physical activity, irregular exercise (less than 30 minutes 1–2 times a week), regular exercise (over 30 minutes 1–2 times a week), and frequent exercise (more than 30 minutes 3 or more times a week).

### Laboratory analyses

Fasting blood was drawn and oral glucose tolerance test (OGTT) was completed by all subjects except those with previously known insulin-treated diabetes mellitus. The subjects were defined to be diabetic if they had a known diabetes mellitus or if their fasting plasma glucose level was 7.0 mmol/l or more, or if their 2-hour plasma glucose was 11.1 mmol/l or more. The concentrations of total cholesterol and triglycerides in the plasma and lipoprotein fractions were determined by enzymatic colorimetric methods (Boehringer Diagnostics, Mannheim GmbH, Germany, catalogue nos. 236691 and 701912), using a Kone Specific, Selective Chemistry Analyzer (Kone Instruments). A detailed description of the laboratory analyses regarding lipid measurements is provided earlier [14].

### Study design and outcome classification

After the baseline clinical examinations and laboratory analyses, the subjects were followed-up for more than 20 years (the end of the follow-up being a death due to any cause or the last day of the year 2014, whichever came first).

CV event included a major coronary heart disease (CHD) event and stroke (excluding subarachnoid hemorrhage [SAH]), whichever of them occurred first. The evidence of CHD was based on the following diagnosis: I20.0, I21, I22 (International Classification of Diseases [ICD-10] and Related Health Problems)/410, 4110 (ICD-8/9) as the main diagnosis (symptom or cause) and I21, I22 (ICD-10)/410 (ICD-8/9) as a first side diagnosis (symptom or cause) or second side diagnosis (symptom or cause) and third side diagnosis (ICD-8/9 only) or if a subject had experienced a coronary artery bypass graft surgery or angioplasty. CHD as a cause of death included I20–I25, I46, R96, R98 (ICD-10)/410–414, 798 (not 7980A) (ICD-8/9) as underlying cause of death or immediate causes of death and I21 or I22 (ICD-10)/410 (ICD-8/9) as first to third contributing cause of death. Stroke (excluding SAH) included I61, I63 (not I636), I64 (ICD -10)/431, 4330A, 4331A, 4339A, 4340A, 4341A, 4349A, 436 (ICD-9)/431 (except 43101, 43191) 433, 434, 436 (ICD-8) as main diagnosis (symptom or cause) or as first or second side diagnosis (symptom or cause) or as third side diagnosis (ICD-8/9 only) or as an underlying cause of death or immediate cause of death or as first to third contributing cause of death.

Information on causes of death and events leading to hospitalization was obtained from the Finnish Causes-of-Death Register and the Hospital Discharge Register.

### Statistical analyses

A total of 1045 subjects were included in the analyses. The baseline characteristics of the study population were compared between males and females, and between those with carotid and/or femoral bruits and those without any audible bruits using independent-samples T test (normally distributed variables), Mann-Whitney U test (skewed variables), or Chi-Square test (categorical variables). Carotid and femoral bruits were computed into three variables: (1) carotid bruit (unilateral or bilateral), (2) femoral bruit (unilateral or bilateral), and (3) carotid or femoral bruit (containing either carotid or femoral bruit or both). Then, the association between the aforementioned variables and mortality (CV deaths and deaths due to all-cause) as well as CV events was analyzed using both Kaplan-Meier and Cox regression survival analyses. The proportional hazard assumption was tested by observing Kaplan-Meier curves (categorical variables) or by creating scatter plots of variable partial residuals as a function of time (continuous variables). Had the Kaplan-Meier curves overlapped or crossed each other or had there been any non-random pattern in the scatter plots, the proportional hazard assumption would not have been met. After univariable analyses, Cox multivariable regression analyses were conducted by adjusting the model with age, sex, systolic blood pressure, diabetes, smoking habits, low-density lipoprotein (LDL) cholesterol, CAD and stroke/TIA. Additional analyses were conducted by excluding those subjects with a known previous CAD event or stroke or transient ischemic attack.

The discrimination and reclassification abilities of carotid and femoral bruits were assessed using the C-index, integrated discrimination index (IDI) and net reclassification index (NRI). When analysing the improvements in reclassification, both continuous and categorical NRI were obtained. The chosen cut-off for categorical NRI was 10%, based on the European guidelines classifying 50–69 years old patients to a very high CV disease risk if their SCORE is 10% or more [6]. First, a baseline risk model predicting CV deaths, CV events and all-cause mortality was created. The baseline model included age, sex, systolic blood pressure, smoking habits, diabetes, and LDL cholesterol. Then, carotid and femoral bruits were added to the model independently and together to see whether they improve the baseline model in predicting the aforementioned endpoints or not.

Statistical analyses were performed using SPSS version 26 (IBM Corp. Released 2019. IBM SPSS Statistics for Windows, Version 26.0. Armonk, NY: IBM Corp) and R Statistics (3.3.1, The R Foundation for Statistical Computing, Vienna, Austria). Statistical significance was set at p<0.05.

## Results

### Study population

The final study population consisted of a total of 1045 subjects, of which 520 (49.8%) were men. The baseline characteristics of the study population are largely identical as reported in our previously published paper including mainly the same set of subjects [15]. The mean (± standard deviation, SD) age of the subjects was 51.3 ± 5.97 years. A total of 709 (67.8%) of the subjects had hypertension and 106 (10.1%) had diabetes at baseline. At baseline, 86 (8.2%) of the subjects had been diagnosed with CAD and 22 (2.1%) with previous stroke or TIA. In a questionnaire about leisure time physical activity, 64 (6.1%) of the subjects reported never encaging in physical activities during leisure time whereas 661 (63.3%) stated to exercise regularly of frequently. The baseline characteristics of our study population are presented in Table 1.

**Table 1:** Characteristics of the study population.

Variable	Carotid bruit	Femoral bruit	No bruit
Number of patients	42	124	891
Age (years)^‡^	55.5 ± 5.6	54.7 ± 5.7	50.7 ± 5.8
BMI (kg/m^2^)	27.5 (25.1–29.7)	26.0 (24.2–30.5)	27.0 (24.5–30.3)
Metabolic syndrome	18 (42.9%)	43 (34.7%)	336 (37.7%)
SBP (mmHg)^†^	157 ± 20.2	152 ± 23.0	148 ± 22.0
DBP (mmHg)	90.4 ± 11.9	88.8 ± 13.2	89.1 ± 12.1
Hypertension	33 (78.6%)	86 (69.4%)	596 (67%)
CAD^†^	10 (23.8%)	17 (13.7%)	65 (7.3%)
Stroke or TIA*	5 (11.9%)	6 (4.8%)	15 (1.7%)
Smoking habits			
Never	18 (42.9%)	49 (39.5%)	427 (47.9%)
Not anymore	12 (28.6%)	25 (28.2%)	207 (23.2%)
Irregularly	0 (0.0%)	0 (0.0%)	13 (1.5%)
Current smoker	12 (28.6%)	40 (32.3%)	244 (27.4%)
Smoking (pack-years)^†^	1.5 (0.0–22.3)	6.5 (0.0–25.3)	1.0 (0.0–15.0)
Alcohol (g/week)	13.5 (0.0–98.8)	24.0 (2.25–88.5)	24.0 (1.75–77.3)
Total cholesterol (mmol/l)	6.14 ± 1.49	5.80 ± 1.13	5.67 ± 1.04
HDL (mmol/l)	1.16 (1.01–1.41)	1.26 (1.06–1.59)	1.30 (1.08–1.56)
LDL (mmol/l)^†^	4.05 ± 1.38	3.65 ± 1.06	3.50 ± 0.92
Triglycerides (mmol/l)	1.47 (1.17–1.96)	1.42 (0.97–1.97)	1.39 (1.00–1.85)
Diabetes	11 (26.2%)	11 (8.9%)	88 (9.9%)
Medication			
ASA*	6 (14.3%)	12 (9.7%)	44 (4.9%)
Anti-hypertensive	23 (54.8%)	56 (45.2%)	470 (52.7%)
Lipid-lowering	5 (11.9%)	2 (1.6%)	24 (2.7%)
Leisure-time physical activity			
No physical activity	2 (4.8%)	5 (4.0%)	57 (6.4%)
Irregular exercise^†^	10 (23.8%)	21 (16.9%)	273 (30.6%)
Regular exercise	14 (33.3%)	30 (24.2%)	289 (32.4%)
Frequent exercise^‡^	16 (38.1%)	67 (54.0%)	255 (28.6%)

The values are means ± SD, medians (1^st^-3^rd^ quartile) or number of patients (% within group). BMI, body mass index; SBP, systolic blood pressure; DBP, diastolic blood pressure; CAD, coronary artery disease; TIA, transient ischemic attack; HDL, high-density lipoprotein; LDL, low-density lipoprotein; ASA, acetylic salicylic acid. Differences in baseline characteristics between those with carotid and/or femoral bruits and those without any bruits were analysed using independent-samples T test (normally distributed variables), Mann-Whitney U test (skewed variables) and Chi-square test (categorical variables).

*p<0.05

†p<0.01

‡p<0.001

42 (4.0%) of the study population had audible carotid bruits, 124 (11.9%) had femoral bruits, 12 (1.1%) had both bruits, and 891 (85.2%) did not have either bruit at baseline. When comparing the baseline characteristics between those with carotid and/or femoral bruits and those without any bruits (Table 1), subjects with audible bruits were older (p<0.01) and had higher systolic blood pressure (p<0.001). Subjects with audible bruits also smoked more (p<0.01), they had higher LDL cholesterol (p<0.01), and they had more CAD (p<0.01) and previous stroke or TIA (p<0.05) at baseline. When comparing the baseline characteristics between males and females (results not shown), males had higher systolic (p<0.001) and diastolic blood pressure (p<0.001), higher triglycerides (p<0.001), higher total and LDL cholesterol (p<0.001 for both), as well as lower HDL cholesterol (p<0.001) compared to females. The prevalence of carotid bruits was similar between genders, but audible femoral bruits were more common in men compared to women (p = 0.049).

### Cardiovascular and all-cause mortality

**Fig 2:**
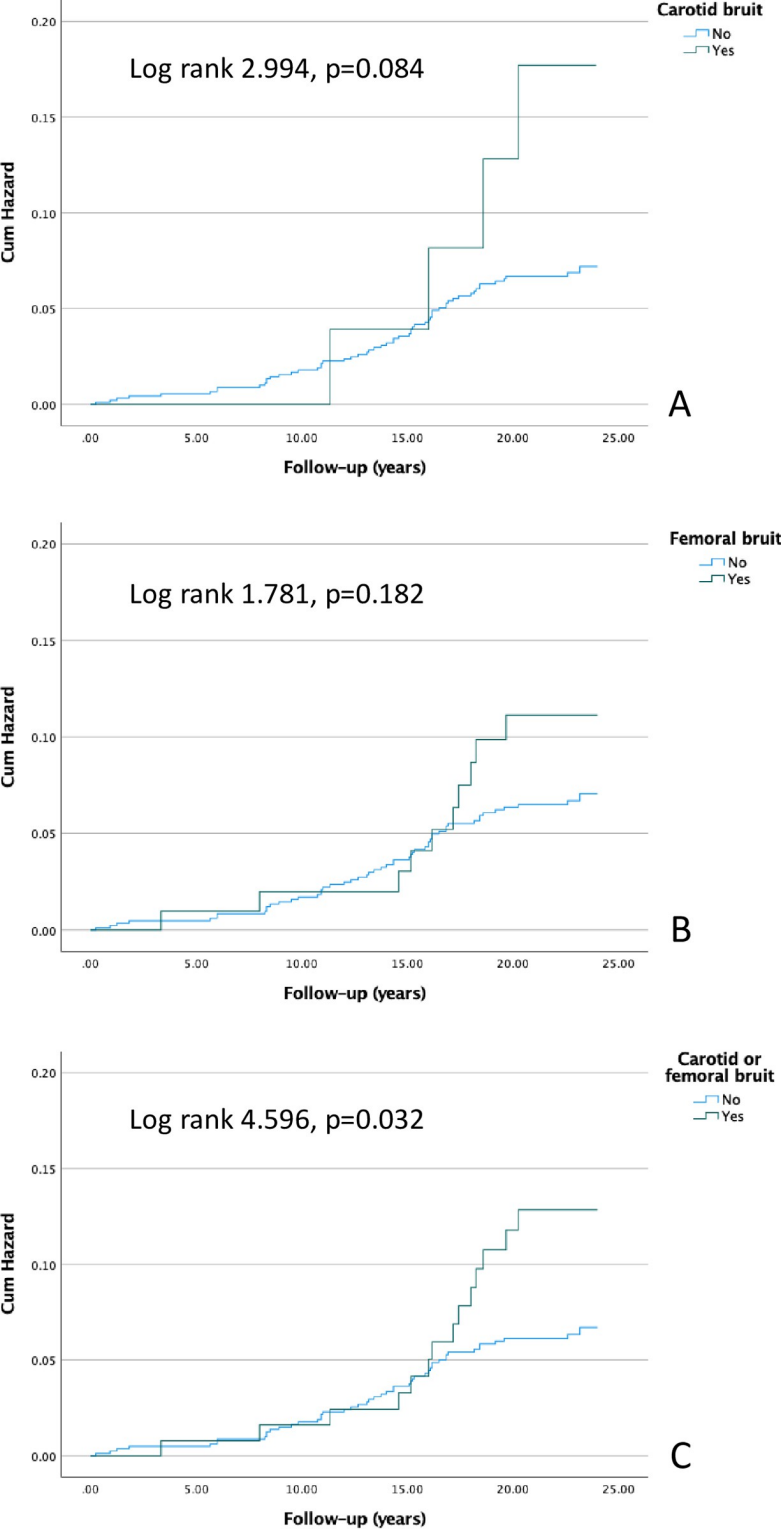
Kaplan-Meier curves presenting the cumulative hazards for cardiovascular deaths in patients with carotid bruit (A), femoral bruit (B), and carotid or femoral bruit (C).

**Fig 3:**
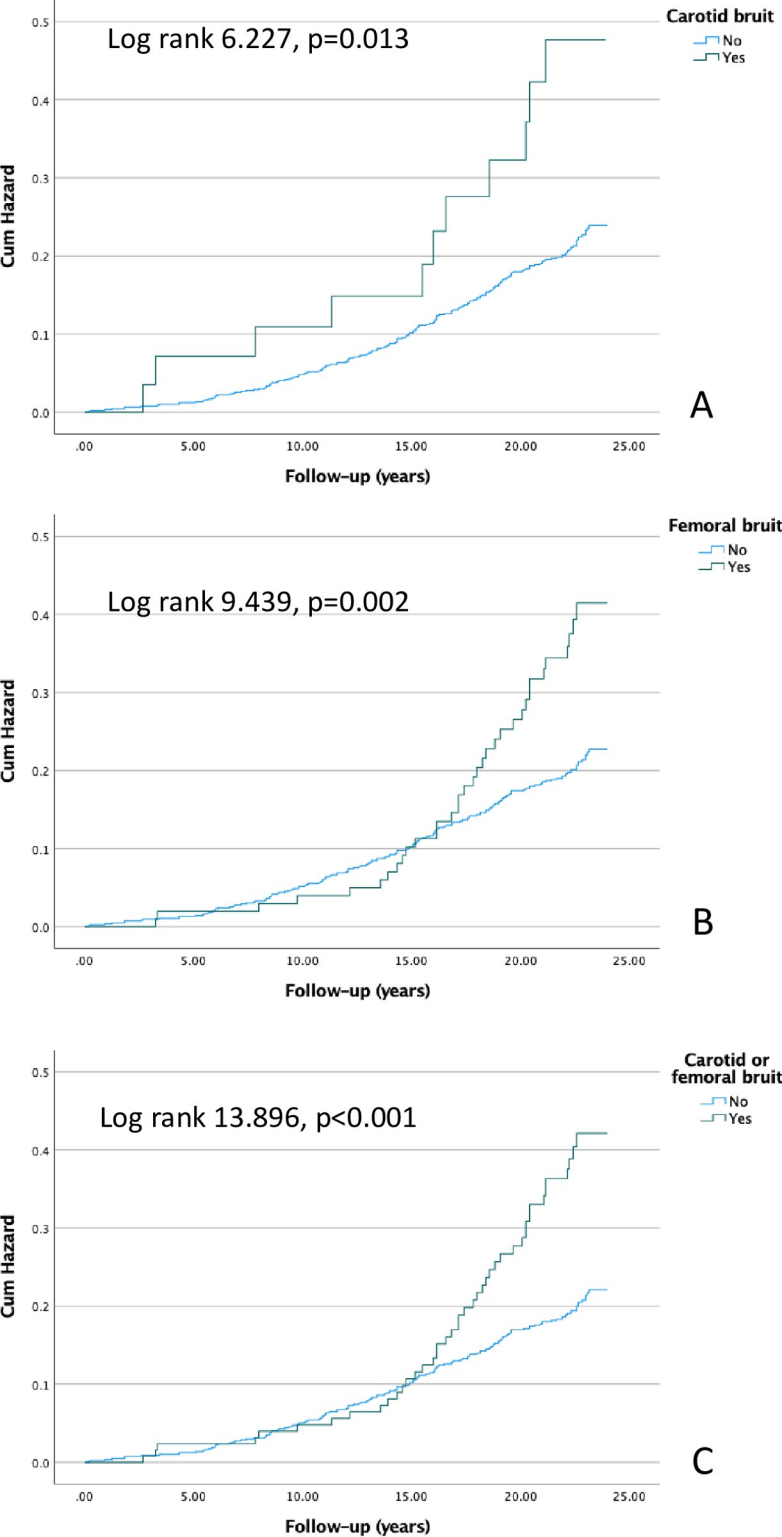
Kaplan-Meier curves presenting the cumulative hazards for all-cause mortality in patients with carotid bruit (A), femoral bruit (B), and carotid or femoral bruit (C).

**Fig 4:**
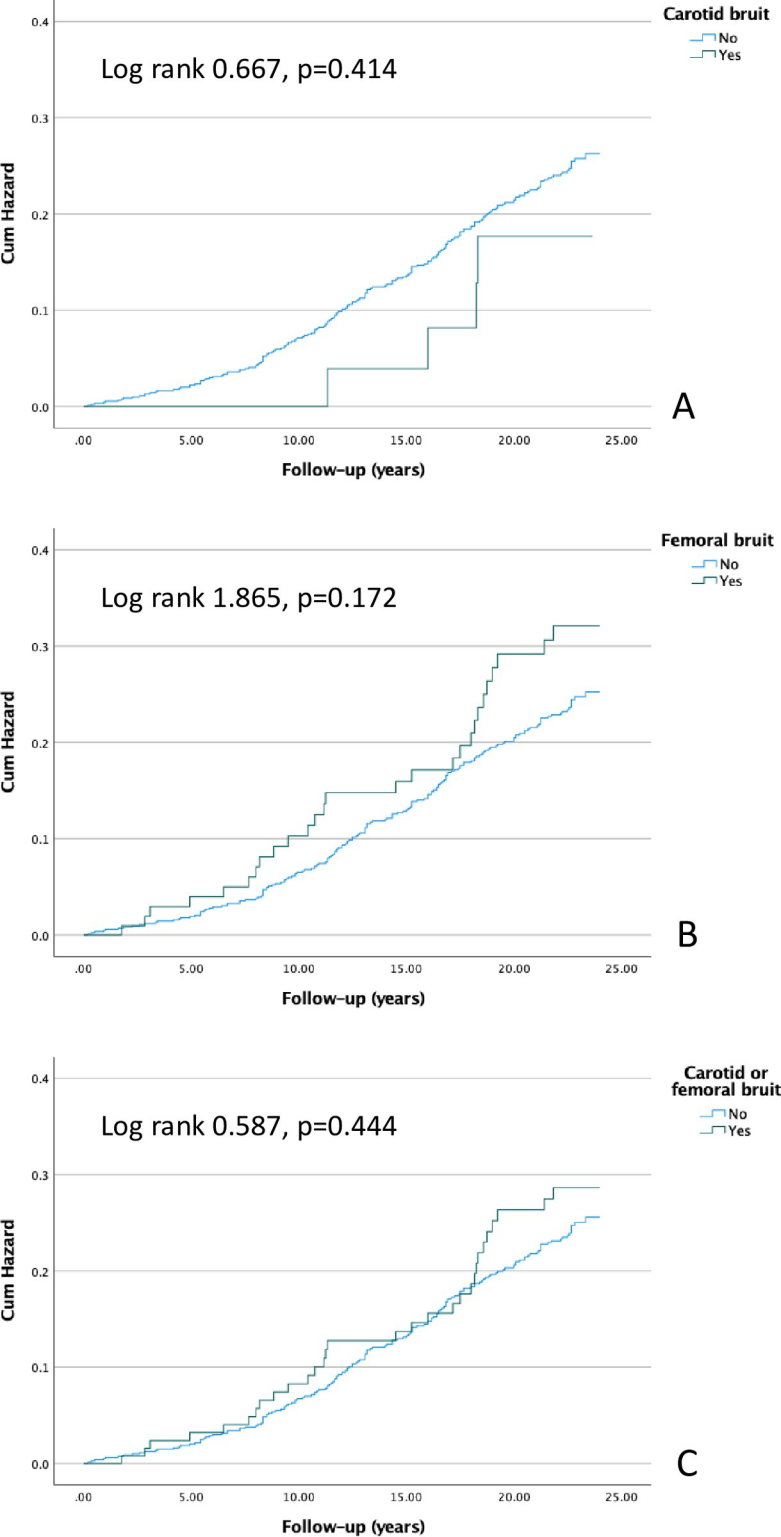
Kaplan-Meier curves presenting the cumulative hazards for cardiovascular events in patients with carotid bruit (A), femoral bruit (B), and carotid or femoral bruit (C).

During a median (1^st^–3^rd^ quartile) follow-up time of 22.6 (22.1–23.3) years, a total of 255 (24.4%) of the subjects suffered a CV event and 241 (23.1%) of the subjects died. Out of those 241 deaths, 82 (34.0%) were categorized to be of CV origin. Kaplan-Meier cumulative hazard curves for CV events, CV deaths and all-cause mortality are presented in Figs 2–4. When all subjects (n = 1045) were included in the analyses, having a carotid bruit indicated a significantly increased risk for CV deaths (HR [95% CI] 4.15 [2.39–8.52], p<0.001) as did also femoral bruits (HR 2.55 [1.54–4.22], p<0.001). The incidence rate of CV deaths was 1.48% (14.8% 10-year incidence) per person-year in individuals with carotid bruits, 0.81% (8.1% 10-year incidence) per person-year in individuals with femoral bruits, and 0.82% (8.2% 10-year incidence) in individuals with either carotid or femoral bruit. Other factors predicting CV deaths were age, HR 1.06 (1.02–1.10), p = 0.005; male gender, HR 2.84 (1.74–4.64), p<0.001; CAD, HR 3.62 (2.11–6.20), p<0.001; previous stroke or TIA, HR 4.27 (1.73–10.6), p = 0.002; systolic blood pressure, HR 1.02 (1.02–1.03), current smoking (compared to never-smokers), HR 4.13 (2.33–7.31), p<0.001; diabetes, HR 3.75 (2.30–6.12), p<0.001 and LDL cholesterol, HR 1.29 (1.03–1.62), p = 0.026. After adjusting for age, sex, systolic blood pressure, smoking habits, diabetes, LDL cholesterol, CAD, and stroke, having a carotid or femoral bruit indicated a 69% increased risk for CV deaths compared to those who did not have any bruits (Table 2).

**Table 2:** Hazard ratios of arterial bruits in predicting cardiovascular deaths and all-cause mortality.

	**Hazard ratio (95% CI)**			
**All subjects (n = 1045)**	**Univariable**	**p-value**	**Multivariable**	**p-value**
**CV events**				
No carotid bruit	1.00		1.00	
Carotid bruit	1.91 (1.15–3.17)	0.012	0.99 (0.57–1.71)	0.967
No femoral bruit	1.00		1.00	
Femoral bruit	1.62 (1.16–2.25)	0.004	1.05 (0.74–1.49)	0.785
No bruits at all	1.00		1.00	
Carotid or femoral bruit	1.49 (1.09–2.04)	0.012	0.95 (0.68–1.32)	0.743
**CV deaths**				
No carotid bruit	1.00		1.00	
Carotid bruit	4.15 (2.39–8.52)	<0.001	2.35 (1.16–4.78)	0.018
No femoral bruit	1.00		1.00	
Femoral bruit	2.55 (1.54–4.22)	<0.001	1.59 (0.92–2.76)	0.100
No bruits at all	1.00		1.00	
Carotid or femoral bruit	2.71 (1.69–4.34)	<0.001	1.69 (1.01–2.80)	0.044
**All-cause mortality**				
No carotid bruit	1.00		1.00	
Carotid bruit	2.59 (1.62–4.14)	<0.001	1.54 (0.94–2.53)	0.088
No femoral bruit	1.00		1.00	
Femoral bruit	2.02 (1.47–2.76)	<0.001	1.33 (0.95–1.85)	0.100
No bruits at all	1.00		1.00	
Carotid or femoral bruit	2.01 (1.50–2.70)	<0.001	1.33 (0.97–1.81)	0.074
	**Hazard ratio (95% CI)**			
**CAD and stroke/TIA patients excluded (n = 941)**	**Univariable**	**p-value**	**Multivariable**	**p-value**
**CV events**				
No carotid bruit	1.00		1.00	
Carotid bruit	0.66 (0.25–1.79)	0.417	0.47 (0.17–1.28)	0.138
No femoral bruit	1.00		1.00	
Femoral bruit	1.33 (0.88–1.99)	0.174	0.94 (0.61–1.44)	0.772
No bruits at all	1.00		1.00	
Carotid or femoral bruit	1.16 (0.79–1.72)	0.444	0.82 (0.55–1.23)	0.341
**CV deaths**				
No carotid bruit	1.00		1.00	
Carotid bruit	2.38 (0.86–6.56)	0.094	1.62 (0.57–4.60)	0.364
No femoral bruit	1.00		1.00	
Femoral bruit	1.58 (0.80–3.11)	0.186	1.06 (0.52–2.19)	0.871
No bruits at all	1.00		1.00	
Carotid or femoral bruit	1.90 (1.05–3.44)	0.035	1.31 (0.70–2.48)	0.401
**All-cause mortality**				
No carotid bruit	1.00		1.00	
Carotid bruit	2.13 (1.16–3.92)	0.015	1.69 (0.90–3.14)	0.100
No femoral bruit	1.00		1.00	
Femoral bruit	1.77 (1.22–2.56)	0.002	1.21 (0.82–1.79)	0.347
No bruits at all	1.00		1.00	
Carotid or femoral bruit	1.88 (1.34–2.64)	<0.001	1.33 (0.93–1.90)	0.118

Survival analyses were conducted by using Cox regression analysis. Each bruit variable was analyzed in a separate Cox model. Multivariable model was adjusted for age, sex, systolic blood pressure, diabetes, smoking habits, LDL cholesterol, CAD, and stroke/TIA. CV, cardiovascular; CI, confidence interval.

If, however, all analyses mentioned above were conducted by excluding the subjects who had had a previous CAD, stroke or TIA event, neither carotid nor femoral bruit was associated with an increased risk of adverse CV outcomes in the multivariable model (Table 2). Moreover, even if subjects with previous CV events were included in the analyses, carotid or femoral bruits were not associated with CV events as an aggregate endpoint (non-fatal + fatal events). Considering the differences between males and females in baseline characteristics, survival analyses were also conducted by stratifying the analyses by gender. Neither carotid nor femoral bruits were associated with the studied endpoints in the multivariable models of these stratified analyses. Adding carotid or femoral bruits in the baseline risk model did not provide any improvements in C-statistic, NRI or IDI parameters.

## Discussion

Our results indicate that audible carotid or femoral bruits are indicating an increased risk for fatal CV events and all-cause mortality in a Finnish middle-aged population not entirely free of previous CV events. In this unselected population, having a carotid or femoral bruits poses nearly a two-fold increased risk for CV death during a 20-year follow-up period. Importantly, neither carotid nor femoral bruits were associated with future CV events if subjects with previous CV events were excluded.

Narrowing of the arterial lumen can obstruct the normal laminar blood flow and cause the blood flow to turn turbulent due to increase in potential energy (pressure) proximal to the stenosis which results in increased kinetic energy (velocity) within the stenosis. If the pressure gradient is sufficient, the turbulent blood flow produces vibrations and an audible bruit [19]. Therefore, basic clinical examination, including auscultation of the vasculature, may provide valuable information on the state of the CV system and reveal a vascular pathology (e.g. arterial plaques) increasing the risk of subsequent CV events.

Both carotid and femoral artery plaques have been documented to predict coronary artery disease [13, 20] and CV events [21, 22]. Since arterial morphological changes can manifest as bruits [19], it seems conceivable that an audible vascular bruit might carry significant prognostic information on individual CV risk. Interestingly, our results suggest that arterial bruits are significant risk factors for fatal CV events only if all of our subjects are included in the analyses (8.2% had CAD and 2.1% had previous stroke). A meta-analysis conducted a decade ago concluded that audible carotid bruits posed over a two-fold risk for CV events and CV deaths [11]. However, as the authors point out themselves, several issues are related to the included studies, not least of which is the lack of information and adjustments of the analyses with common CV risk factors (e.g., hyperlipidemia, diabetes, hypertension). Although in light of the meta-analysis by Pickett et al. carotid bruits appear as an appealing surrogate marker for CV event risk, they might not provide much useful information in a population not entirely free of previous CV events, as our findings would suggest. That being said, it needs to be remembered that the overall number of subjects with audible bruits in our study was low, and, consequently, after excluding those with previous CAD and stroke events, the statistical power of our analyses was greatly reduced (only 4 CV events occurred in those with carotid bruit, for example). This suggests the limited usefulness of peripheral arterial bruits to be used in CV risk estimation in a pure primary prevention setting.

Unlike carotid bruits, there have been much less clinical research on the association between femoral bruits and adverse CV outcomes, which is interesting given the previous research showing the extent and importance of subclinical atherosclerosis in non-coronary locations, namely abdominal aorta and femoral arteries [13, 23]. One article from the 1980’s reported that abnormal pulse examination (femoral bruit or abnormal posterior tibial pulse) posed a relative risk of 3.4 for mortality in a 4-year follow-up compared to those with normal pulse examination [24]. Worth noting is that half of their study population consisted of patients having dyslipidemia or taking lipid-lowering medications. A more recent article by Cournot et al. (2009), consisting of participants with similar baseline characteristics as in our study, demonstrated that after adjusting for the Framingham Risk Score, femoral bruits predicted coronary events whereas carotid bruits did not [12]. The findings of Cournot et al. and the possible superiority of femoral bruits over carotid bruits in predicting CV events are supported by the earlier evidence (in all-men population) showing that femoral plaques were more accurate predictors of coronary artery calcium compared to carotid plaques [13]. On the contrary to these previous studies, we did not detect any statistical association in the multivariable analyses between femoral bruits and adverse CV outcomes.

The association between non-coronary atherosclerosis (e.g., carotid and lower limb arteries) and coronary artery disease, and the evidence of the increase in CV event rates associated with multifocal atherosclerosis [25, 26] highlights the importance of thorough clinical examination when assessing the overall CV risk profile of the patient. Unfortunately, when it comes to carotid bruits, for example, previous studies have reported poor sensitivity for audible bruits in detecting clinically significant stenoses [27, 28]. Moreover, the lack of data on cost-effectiveness and the potential harms associated to false positives are two major reasons why the use of clinical or imaging examinations for screening of CV risk in asymptomatic patients are not generally endorsed [29, 30].

In light of the poor accuracy of arterial bruits in detecting actual stenoses and the current consensus on the stenosis management, detecting an audible bruit during clinical examination should not necessarily lead to further examination or treatments aimed towards the supposed stenosis. Furthermore, since our findings suggest that carotid or femoral bruits do not associate with CV events if individuals with previous CV events are excluded, it is difficult to see their clinical utility in clinical decision-making when it comes to primary prevention of CV diseases. Even though our results demonstrate that individuals with carotid bruits have a 14.8%, and with femoral bruits an 8.2% 10-year risk of fatal CV events, the performed survival analyses indicate that audible bruits do not provide information beyond traditional CV risk factors in a population free of previous CV events. On the contrary, as per our results, carotid bruits indicate a doubled risk of fatal CV events in a population where previous CV events exist, which would suggest some potential of carotid bruits in assessing the risk of recurrent CV events.

There are some limitations related to our study. First, our study population is relatively small which reduces the statistical power of our analyses. Another obvious limitation in our study is that the exposure variables are based only on a single baseline clinical examination, and the statistical analyses rely on the assumption that the baseline characteristics remain constant throughout the follow-up period, which is unlikely the case. The auscultations were performed by two physicians: one auscultated carotid and femoral arteries of every male participant and the other every female participant. Since the variability between the two examiners was not tested, it is difficult to draw conclusions of the between-gender prevalence of audible bruits. Also, due to relatively high blood pressure criteria used to define subjects as hypertensive during the recruitment phase of the study in the 1990s [14], a larger proportion of our study population was hypertensive (67.8%) in today’s hypertension criteria than was originally intended.

## Conclusions

The present study is currently the longest prospective setting exploring the role of carotid and femoral bruits in predicting adverse CV events in the future. With the limitations related to our study in mind, our results suggest that neither carotid nor femoral bruits provide clinically useful information in primary prevention setting of CV diseases in a Scandinavian middle-aged population. Audible bruits may carry prognostic information in an unselected population where recurrent CV events may occur.
